# The role of Th17 cells in inflammatory bowel disease and the research progress

**DOI:** 10.3389/fimmu.2022.1055914

**Published:** 2023-01-09

**Authors:** Lu Chen, Guangcong Ruan, Yi Cheng, Ailin Yi, Dongfeng Chen, Yanling Wei

**Affiliations:** Department of Gastroenterology, Chongqing Key Laboratory of Digestive Malignancies, Daping Hospital, Army Medical University (Third Military Medical University), Chongqing, China

**Keywords:** inflammatory bowel disease, T helper 17 cells, inflammatory, intestinal fibrosis, gut microbiota

## Abstract

Th17 cells play an important role in the abnormal immune response in inflammatory bowel disease (IBD) and are involved in the development and progression of inflammation and fibrosis. An increasing amount of data has shown that gut microbes are important parts of intestinal immunity and regulators of Th17 cellular immunity. Th17 cell differentiation is regulated by intestinal bacteria and cytokines, and Th17 cells regulate the intestinal mucosal immune microenvironment by secreting cytokines, such as IL-17, IL-21, and IL-26. Solid evidence showed that, regarding the treatment of IBD by targeting Th17 cells, the therapeutic effect of different biological agents varies greatly. Fecal bacteria transplantation (FMT) in the treatment of IBD has been a popular research topic in recent years and is safe and effective with few side effects. To further understand the role of Th17 cells in the progression of IBD and associated therapeutic prospects, this review will discuss the progress of related research on Th17 cells in IBD by focusing on the interaction and immune regulation between Th17 cells and gut microbiota.

## 1 Introduction

Inflammatory bowel disease (IBD) is a chronic inflammatory gastrointestinal disease whose etiology and pathogenesis are not completely understood. IBD can be divided into three categories, Crohn’s disease (CD), ulcerative colitis (UC), and inflammatory bowel disease unclassified (IBDU). It is generally believed that IBD was induced by environmental factors acting on genetically susceptible populations, where the body initiates an abnormal immune response with the involvement of gut microbes, thereby leading to pathological processes such as intestinal mucosal barrier damage and inflammatory cell infiltration. Activated CD4^+^ T cells are the dominant effector cells mediating abnormal immune responses and subsequent inflammation in the intestinal mucosa. Previous studies have proved the relevance of CD and UC to T helper (Th)1- and Th2-mediated immune responses (1). Recent researches have affirmed that Th17 cells also play an essential role in the abnormal immune response in IBD pathophysiology (2, 3). Th17 cells were first described as a discrete subpopulation of Th0 cells in 2005 (4). They are differentiated from naive CD4^+^ T cells under the regulation of antigen-presenting cells (APCs) and their secreted cytokines, which specifically secrete interleukin (IL)-17 and cause tissue inflammation (4, 5).

The expression of chemokine receptor 6 (CCR6) on the surface of Th17 cells allows them to migrate to specific intestinal tissue targets in the presence of the CCR6 ligand chemokine (C-C motif) ligand 20 (CCL20) (6). Normally, Th17 cells and their secreted cytokines play essential roles in fighting infections and retaining intestinal immune homeostasis (7). When dysregulated, the hyperproliferation and activation of Th17 cells induce abnormal immune responses and lead to various autoimmune diseases, including IBD (8). As one of the essential members of the "gut microecosystems", the gut microbiota is involved in the regulation of Th17 cells and the progression of IBD. To further understand the role of Th17 cells in IBD progression and their therapeutic potential, this article will review the progress of Th17 cell research, their role in IBD, and their association with gut microbiota.

## 2 The role of Th17 cells and their secreted cytokines in the pathogenesis of IBD

**Figure 1:**
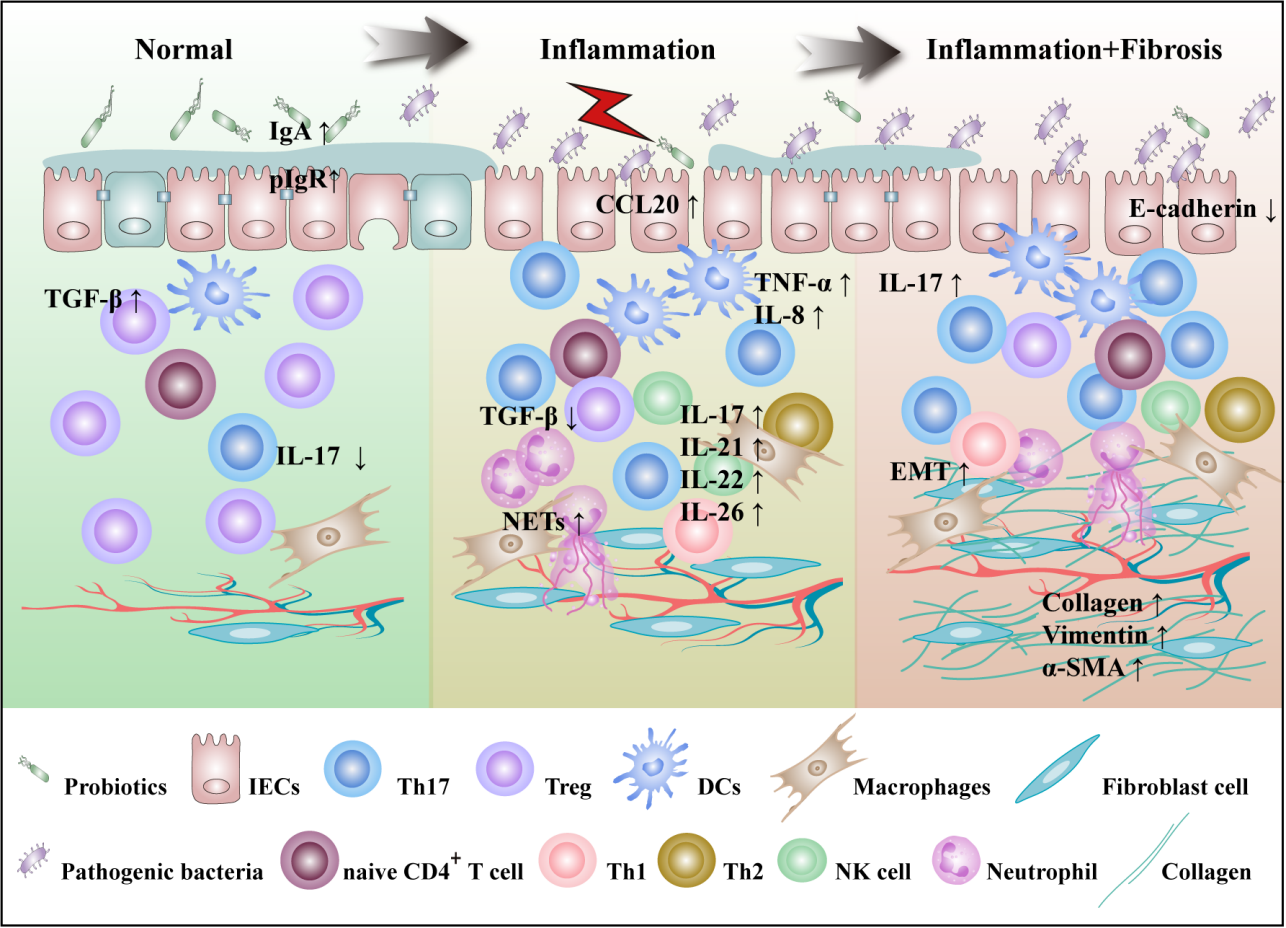
Th17 cells are involved in the pathogenesis of IBD. The pathogenesis of IBD is characterized by gut microbes imbalance, mucosal damage, inflammation and fibrosis. In the normal intestinal microecosystem, Th17 cells and Tregs are in dynamic balance. IL-17 secreted by Th17 cells inhibits the colonization of pathogenic bacteria by targeting IECs that enhance the secretion of IgA and antimicrobial peptides, while Tregs inhibit excessive immunity of T cells. Under the influence of external stimuli and susceptibility genes, imbalances in gut microbiota lead to a decrease in SCFA-producing bacteria, including *R.intestinalis*, and an increase in pathogenic bacteria, including *SFB*. DCs are activated to produce cytokines such as IL-6, IL-23, and TGF-β. Th17 cells migrate by CCL20 secreted by IECs to the site of inflammation, where they activate and secrete a large number of cytokines to participate in the progression of inflammation. At the same time, IL-17 induces IECs to express IL-8, which recruits a large number of neutrophils and NETs at sites of inflammation, positively regulating Th17 cell feedback. IL-21 is involved in the regulation of Th17 cell differentiation in an autocrine manner. IL-22 secretion by Th17 cells was shown to act on NK cells and goblet cells and promote goblet cell mucus production. With the continuous progression of inflammation, IL-17 can induce fibroblasts to secrete ECM and promote the progression of intestinal fibrosis. *R.intestinalis*, *Roseburia intestinalis*; NETs, neutrophil extracellular traps; ECM, extracellular matrix.

Th17 cells are predominantly located on the lamina propria of intestinal lumen and defend the host from invasion by pathogenic microorganisms. Abundant mucosal Th17 cells are common in IBD patients and animal models. Compared with IBD remission stage, Th17 cells increased in peripheral blood and intestinal mucosa at the active stage. These cells are principally involved in disease progression by secreting cytokines such as IL-17A, IL-17F, IL-21, IL-22, and IL-26. Hereinafter, we will elaborate on the role of Th17 cells from the perspective of the disease occurrence and progression of IBD, as shown in **Figure 1**.

### 2.1 Th17 cells in the maintenance of intestinal homeostasis

Th17 cells are implicated in gastrointestinal immune responses. Under homeostasis conditions, "gut microecosystems" are in dynamic balance, under the joint action of intestinal microorganisms, cytokine signals, and costimulatory signals, Th17 cells and regulatory T cells (Tregs) are in dynamic balance, they collaboratively maintain the intestinal immune microenvironment homeostasis and intestinal health. Th17 cells express and secrete small quantities of IL-17 and IL-22 to promote epithelial cell proliferation and upregulate antimicrobial peptides and tight junction proteins expression, thus protecting the intestinal mucosa from infections by various pathogens.

IL-17 can promote intestinal epithelial cells (IECs) proliferation, increase the polymeric Ig receptor (pIgR) expression, promote intestinal IgA secretion, and secrete a variety of antimicrobial peptides to accelerate the healing of intestinal mucosal injury, thereby enhancing the intestinal barrier function (9). By binding to Th1 cell surface receptors, IL-17 also inhibits from secreting IL-23R, IFN-γ, IL-12Rp2, and other proinflammatory factors, thereby inhibiting their immune regulation (10). IL-17 inhibits intestinal inflammation by upregulating the number of atypical M2 macrophage subsets (11). IL-22 promotes signal transducer and activator of transcription 3 (STAT3) activation in colonic epithelial cells and induces mucus-related molecule expression and secretion of mucus goblet cell repair products (12). Upregulation of TGF-β expression in the gut microenvironment mediates Th17 cells transdifferentiation into Tregs. Tregs express the anti-inflammatory factor TGF-β, which can inhibit the excessive immune response of effector T cells and protect intestinal tissue from injury.

### 2.2 Th17 cells in inflammatory bowel diseases

#### 2.2.1 Th17 cells with inflammation

Under the regulation of susceptibility genes and environmental hazards, the bacterial diversity, composition, and abundance of the gut microbiota changed. Pathogenic bacteria promote colonization and infection by breaking down mucins as nutrients, releasing toxic gases and secreting enzymes to disrupt mucosal barriers. The number of Th17 cells with upregulated IL-17 expression increased in inflammation foci in IBD, while the number of Tregs were decreased and the expression of TGF-β was downregulated (13).

Th17 cells promote inflammation primarily through the secretion of cytokines. IL-17 can exert proinflammatory effects through the following pathways: IL-17 alone or in conjunction with tumor necrosis factor (TNF)-α acts on IECs to promote the secretion of inflammatory mediators, chemokines, and proteases (e.g., IL-6, IL-8, inducible nitric oxide synthase (iNOS), CXCL8, TNF-α, matrix metalloproteinase (MMP) and granulocyte-macrophage colony-stimulating factor (GM-CSF)). These factors induce inflammation and promote the enlisting, activation and movement of neutrophil to target tissues, causing intestinal mucosal injury (14). Additionally, IL-17 can synergistically activate the NF-κB, ERK1/2, and p38 signaling pathways with TNF-α to induce IL-17C secretion from enteric neuroendocrine cells and goblet cells and promote the expression of the Th17 chemokine CCL20 in IECs. The upregulation of CCL20 expression and the occurrence of neutrophil production of neutrophil extracellular traps (NETs) further promotes the recruitment and activation of Th17 cells, leading to aggravation of the inflammatory response. IL-26 amplifies the aberrant inflammatory immune response; upregulates IL-6 and IL-8 expression through the STAT1, STAT3, PI3K/Akt, and MAPK signaling pathways; induces the adhesion molecules MMP-8 and MMP-9 expression; and activates macrophages (15, 16).

CD and UC are caused by Th1 and Th2 cells, respectively. Th17 cells have the ability to redifferentiate into Th1 or Th2 cells. IL-12 induces a rapid transformation of the Th1-like phenotype by restimulating the Th17 precursor (17). IFNγ secreted by progeny of Th17 precursors is pathogenic in some autoimmune models. In addition, IL-1β, IL-4, and IL-23 induced naive CD4^+^ T cells to differentiate into double-positive Th2-Th17 cells (18). Notably, IL-4 repolarizes Th17 precursors to a Th2 phenotype. CD161 is a specific marker for Th17 progenitor cells that are not expressed by traditional Th1 and Th2 cell populations, that can be used to distinguish whether Th1 or Th2 cells are transdifferentiated from Th17 cells.

#### 2.2.2 Th17 cells with fibrosis

Intestinal fibrosis, which is usually the consequence of chronic inflammation, is a common outcome in IBD patients, resulting in internal strictures, progressive structural deformation and loss of function. More than 50% of CD patients develop penetrations or stenosis due to fibrous stenosis, which in most cases requires surgery. During UC progression, the frequency of stricture formation varies from 3.2% to 11.2%, with 68% of stenosis cases occurring in the rectum (19). Surgery is the only option available to improve intestinal stenosis and obstruction, but recurrence after surgery is extremely high. The pathogenesis of intestinal fibrosis points to a new concept of the central role of Th17 and IL-17 in the immune response, although their accurate roles in chronic inflammation and IBD are controversial (20).

Th17 cells have been demonstrated to promote a fibrotic phenotype in a variety of diseases, including oral submucosal lesions, vascular fibrosis, colon fibrosis and renal fibrosis (21–24). IL-17A promotes fibrosis by targeting different cells in different tissues, including hepatic stellate cells (HSCs) and Kupffer cells in the liver, and induces cardiac fibrosis by promoting cardiac fibroblast proliferation and HSC activation. In the murine model, inhibition of IL-23 and IL-17 attenuated 2,4,6-trinitrobenzene sulfonic acid (TNBS)-induced intestinal fibrosis. Following persistent intestinal inflammation, IL-17A promotes the production of extracellular matrix (ECM) components and reduces the migratory capacity of myofibroblasts (25). IL-17A overexpression is observed in CD stenotic surgical samples compared to nonstenotic regions from the same individuals and healthy intestinal samples from control individuals (19). IL-17 is overexpressed in stenosis in CD fibrosis, and IL-17A promotes fibroblast proliferation and survival through metabolic reprogramming (26), and induces epithelial-mesenchymal transition (EMT) in IECs, resulting in raised expression of vimentin, snail and α-SMA and lessened E-cadherin expression (27). Increasing evidence indicates that IL-22 is a key molecule in cell proliferation, mucosal healing and tissue repair in relieving fibrosis (20). It is worth noting that some murine CD models exhibit exacerbated midgut fibrosis when the IL-23/IL-22 signaling pathway is upregulated (28).

Th17/IL-17 is proposed to be the core of tissue fibrosis, which promotes myofibroblast proliferation and collagen deposition by connecting established profibrotic molecules and pathways such as TGF-β, various growth factors, and novel profibrotic mediators TL1A/DR3 and Ang-II, thus leading to intestinal fibrosis. Further research on the role of IL-22 and IL-26 in IBD fibrosis is needed.

## 3 Differentiation and regulation of Th17 cells

**Figure 2:**
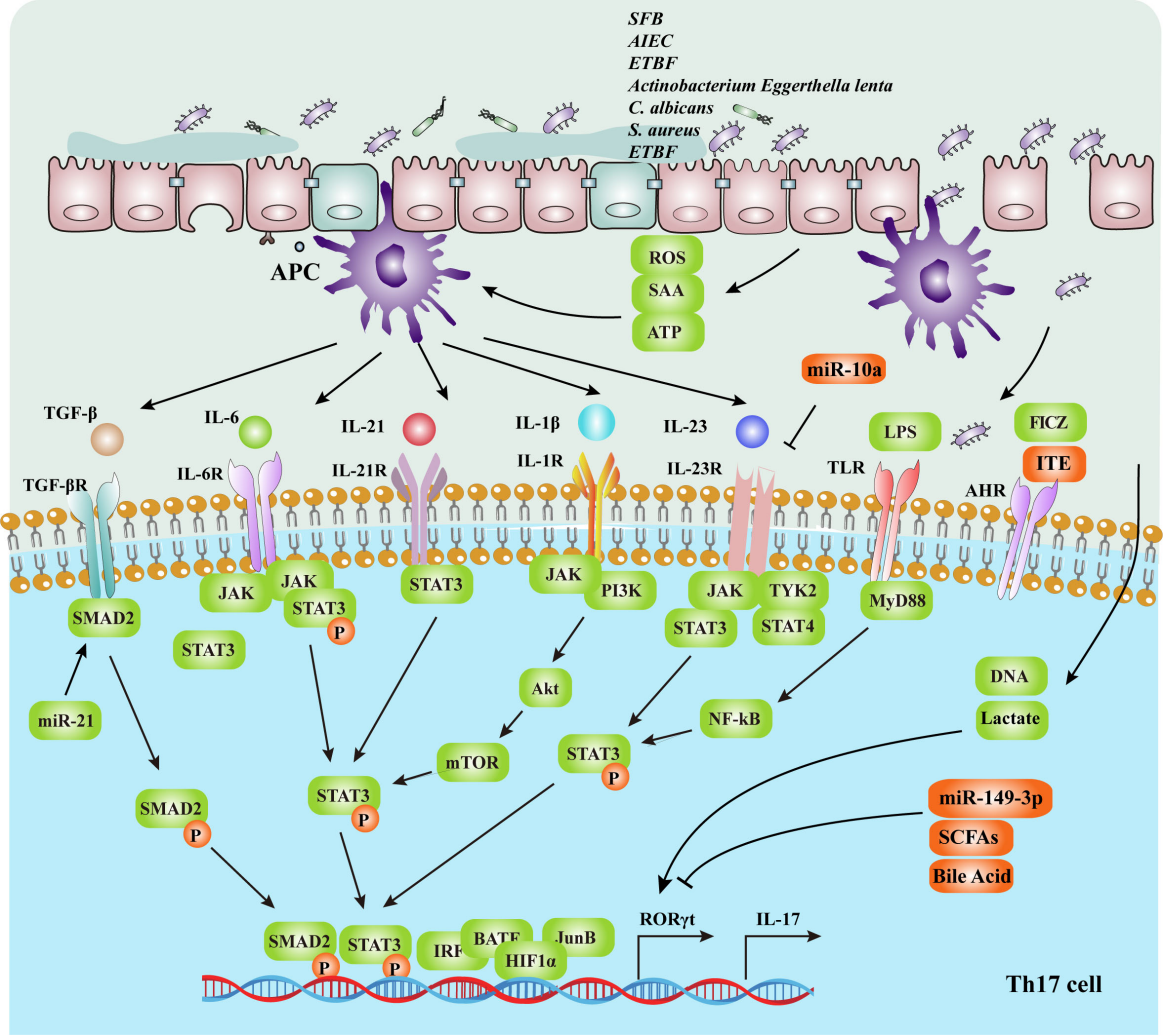
The intestinal mucosal microenvironment, composed of immune cells, cytokines, transcription factors and gut microbiota, regulates Th17 cell differentiation. The gut microbiota activates APCs and upregulates the expression of IL-6, IL-1β and IL-23. IL-6, IL-22, TGF-β, IL-1β and IL-23 can promote the differentiation of Th17 cells by upregulating the expression of the RORγt gene, which depends on the STAT3 signaling pathway. RORγt is a Th17 cell-specific transcription factor that directly affects the differentiation of Th17 cells. In addition, The gut microbiota regulates the differentiation of CD4^+^ T cells through metabolites and energy metabolism, such as LPS, SCFAs and secondary bile acid. Upregulation of the transcription factors IRF4, BATF, HIF-1 and JunB promotes the expression of the RORγt gene. Activation of the TLRs signaling pathways also promotes Th17 proliferation, thereby promoting naive T-cell differentiation into Th17 cells and IL-17 secretion. *SFB, segmented filamentous bacteria; AIEC, adherent invasive Escherichia coli; ETBF, enterotoxigenic Bacteroides fragilis*; SCFAs, short-chain fatty acids; SAA, serum amyloid A; APCs, antigen-presenting cells; Th17, T helper 17 cells; RORγt, retinoic acid receptor-related orphan nuclear receptor γt.

The differentiation of Th17 cells is influenced by gut microbes, metabolic pathways, cytokines, transcription factors, and signal transduction pathways. The specific mechanisms are illustrated in **Figure 2** and **Table 1**. Gut microbes promote naive CD4^+^ T cells differentiation into Th17 cells by upregulating the expression of STAT3 and retinoic acid receptor-related orphan nuclear receptor γt (RORγt) by inducing APCs to secrete cytokines as IL-6 and IL-23 or by metabolites and direct induction (62).

**Table 1:** Regulation of Th17 differentiation.

Regulatory levels	Regulatory factors	Regulatory mechanism	Ref
Gut microbiota and its metabolites	*SFB*	Activates DCs to mediate Th17 cell differentiation Stimulates epithelial cells to secrete SAA protein, cooperates with STAT3-activating cytokines, and mediates Th17 cell differentiation	(29)
	*AIEC*	Sensitizes Th17 cells to produce IL-17	(30)
	*ETBF*	Regulates Th17-cell differentiation *via* miR-149-3p	(31)
	A*ctinobacterium Eggerthella lenta*	Induces intestinal Th17 activation via cardiac glycoside reductase 2 (Cgr2) enzyme	(32)
	*C. albicans*	Promotes the secretion of IgG1 and IL-6 from B cells via TLR2 activation	(33)
	*S. aureus*	Sensitizes Th17 cells to produce IL-17	(34)
	*ETBF*	Downregulates miR-149-3p and facilitates Th17 cell differentiation	(31)
	miR-21	Promotes Th17 cell development by targeting and inhibiting SMAD-7	(35)
	miR-10a	Inhibits Th17 cells in the gut via IL-12/IL-23p40 signaling pathway	(35)
	LPS	Induces Th17 cell differentiation	(36)
	ATP	Activates CD70^high^CD11c^low^ lamina propria cells via P2X and P2Y receptors, thus enhancing the secretion of IL-1β, IL-6 and IL-23 and promoting Th17 cell development	(37)
	FICZ	Upregulates IL-17 and IL-22 production	(38)
	ITE	Induces Tregs	(38)
	SCFAs	Inhibits IL-17 expression and promotes Foxp3 gene expression	(39, 40)
	3-oxoLCA	Inhibits Th17 cell differentiation by directly binding to RORγt	(41, 42)
	isoalloLCA	Increases Foxp3 expression by producing mROS, resulting in promoting Treg cell differention	(41, 42)
Metabolic pathway	Polyamine metabolism	Spermidine, a metabolite of polyamine metabolism, is the basis of faithful Th lineage commitment as a substrate for eIF5A hypination	(43)
	Lipid oxidative metabolism	High-fat diet promotes Th17 cell differentiation and similar to acetyl-CoA carboxylase	(44)
	Glycolytic programs	Regulates Th17 differentiation through the transcription factor HIF1α/mTOR signaling pathway	(45)
	Lactate	Inhibits the proliferation of Th17 cells and increases IL-17A expression	(46)
Cytokines	IL-6	Activates JAK2-STAT3 signaling pathway, upregulates the expression of RORγt gene and promotes Th17 cell differentiation	(47)
	TGF-β	Activates Smad2 signaling pathway, promotes Th17 cell differentiation and promotes the emergence of IL-22-producing	(48)
	IL-23	Promotes Th17 cell differentiation and is a key factor perpetuating Th17 cell activation and cytokine production	(49)
	IL-21	Promotes the differentiation of Th17 cells and increases expression and secretion of IL-17A and IL-22 by upregulating RORγt expression, and inhibits Tregs differentiation by downregulating Foxp3 expression	(50)
	IL-1β	Promotes Th17 polarization via the IL1R/PI3K-mTOR signaling pathway	(51)
Transcription factors	STAT3	Binds to the promoter of IL-17 and directly controls the expression of the transcription factor RORγt for Th17 differentiation transcription factors	(52)
	RORγt	Promotes IL-17 gene transcription by directly binding to the IL-17 gene promoter	(47)
	IRF4	Promotes Th17 cell differentiation by interacting with BATF–JUN heterodimers to bind to AICEs or cooperating with the transcription factor STAT3	(53)
	BATF	BATF knockout inhibits the differentiation of T helper Th17 cells	(54)
	JunB	In collaboration with basic leucine zipper BATF, regulates the expression of Th17-related genes	(55)
	HIF-1	Transcribes and activates RORγt, and promotes Th17 cell development when recruitment to the IL-17 promoter via tertiary complex formation with p300 and RORγt	(56)
Toll-like receptors	TLR2	Causes STAT3 phosphorylation	(57)
	TLR3	Induces DC cells to produce IL-23 and synthesis of IL-17A and IL-21	(58)
	TLR4	Promotes Th17 differentiation by downregulating the expression of miR-30a, activating RelA	(59)
	TLR7	Inhibits Th17 cell differentiation and IL-17 production in established Th17 cells	(60)
	TLR8	Mediates the expression of IL-23 in neutrophils	(61)

### 3.1 Regulation of Th17 cell differentiation by gut microbiota and its metabolites

The microbiota is thought to influence the development and progression of IBD. In previous studies, intestinal Th17 cells increased and mediated colonic inflammation in germ-free mice by transferring gut microbes from IBD mice (63). Bacterial activation of Th17 cells exacerbates autoimmune responses in IBD. Adherent invasive *Escherichia coli* (*AIEC*) induces an active Th17 response in mice, increasing transmural inflammation and fibrosis (30). Existing studies have suggested that the gut microbiota can induce Th17 cell differentiation either directly through contact with immune cells or through metabolites indirectly. A complex microenvironment of commensal flora drives IL-23 production by IECs or dendritic cells (DCs) in the ileum propria (64, 65). Microbial antigens are able to stimulate naive T cells to differentiate into Th17 cells when combined with TLR5 on the surface of CD11c^hi^CD11b^hi^ lamina propria DCs.

*Segmented filamentous bacteria* (*SFB*) can stimulate reactive oxygen species (ROS) production and use microbial adhesion-triggered endocytosis to transfer antigenic proteins into IECs, thus upregulating the secretion of IL-1β and IL-23 and inducing Th17 cell differentiation. *SFB* colonization induces the production of SAA in terminal ilea, and SAA may induce DCs to secrete IL-23 to maintain Th17 survival and activation as well as IL-17 expression, thereby leading to aggravation of IBD while maintaining chronic intestinal inflammation (29). Notably, *SFB* expansion is limited by Th17-derived IL-17A in turn through upregulating ROS and antimicrobial peptide production. The A*ctinobacterium Eggerthella lenta* expresses the cardiac glycoside reductase 2 (Cgr2) enzyme. Cgr2 activates intestinal Th17 cells to upregulate IL-17A expression in a RORc-dependent manner (32). *Candida albicans* (*C. albicans*) is usually a beneficial species of the human gut microbiota, which can become pathogenic in immunocompromised hosts. *C. albicans* hyphae directly activate B cells to express IgG1 and IL-6, leading to the induction of Th17 cell differentiation and eventual bacterial clearance (33). *Staphylococcus aureus* (*S. aureus*)-specific Th17 cells express and secrete IL-17, along with IL-10 after restimulation (34).

Commensal bacteria affect Th17 cell differentiation by regulating miRNAs, such as miR-149-3p, miR21 and miR-10a. *Enterotoxigenic Bacteroides fragilis (ETBF)* downregulated miR-149-3p. MiR-149-3p can be released from exosomes and mediate enteritis by regulating the differentiation of Th17 cells (31). MiR-21 promotes Th17 cell development by targeting and inhibiting SMAD-7, which negatively regulates TGF-β signaling (35). MiR-10a is downregulated by microorganisms through a MyD88-dependent pathway that promotes Th17 cells in the gut via the IL-12/IL-23p40 signaling pathway (35). Gut microbes and miRNAs can form a regulatory network with the immune system.

Enterobacterial metabolites are important players in immune regulation. Lipopolysaccharide (LPS) is a bacterial surface glycolipid produced by *gram-negative bacteria* that induces Th17 cell differentiation and mediates chronic and acute inflammatory responses (36). Symbiotic bacteria provide high concentrations of adenosine triphosphate (ATP). Gut microbiota-derived ATP activates CD70^high^CD11c^low^ lamina propria cells through P2X and P2Y receptors, thus enhancing the secretion of IL-1β, IL-6 and IL-23 and promoting a Th17-polarizing microenvironment (37). 2-(1’H-indole-3’carbonyl)-thiazole-4-carboxylic acid methyl ester (ITE) and 6-formylindolo (3-2b) carbazole (FICZ) are metabolically produced by tryptophanase-positive bacteria such as Lactobacillus reuteri and are involved in immune regulation through the activation of aryl hydrocarbon receptors (AHRs). FICZ upregulates IL-17 and IL-22 production in CD4^+^ T cells, and ITE induces Tregs (38).

In addition, *Clostridium* can inhibit the release of the proinflammatory factor IL-17 through short-chain fatty acids (SCFAs), thereby reducing the differentiation of Th1 and Th17 cells (39, 40). The mechanism by which SCFAs regulate T-cell differentiation includes the mTOR pathway, G-protein-coupled receptor signaling, and epigenome remodeling to promote Treg cell differentiation. For example, butyrate promotes Treg cell differentiation by acetylating histone H3 at the forkhead box protein P3 (Foxp3) locus, and inhibits Th17 cell differentiation by inhibiting C-myc and HDAC-related metabolism. Microorganisms regulate Th17 cell differentiation through bile acid metabolism (41, 66, 67). Saiyu Hang et al. discovered that two different lithocholic acids (LCA) derivatives, isoalloLCA and 3-oxoLCA, act as T-cell regulators in mice to regulate Th17-cell differentiation (41, 42). IsoalloLCA promotes Treg cell differentiation by producing mitochondrial ROS (mROS), and 3-OxoLCA inhibits Th17 cell differentiation by directly binding to RORγt. In a related study, Riping Xiao et al. studied the binding affinity of bile acid metabolites to the IL-17A transcription factor RORγt by synthesizing the sulfated product of LCA, LCA-3-sulfate (LCA-3-S). LCA-3-S specifically targets RORγt to inhibit Th17 cell differentiation, a process that does not affect Th1, Th2, and Treg cell differentiation (68).

The intestinal contents combined with the intestinal immune microenvironment constitute the "gut microecosystems". In the gut microecosystem, the gut microbes occupies a considerable proportion, participates in the regulation of the immune microenvironment and is a valued participant in the regulation of Th17 cell differentiation. Unfortunately, the gut microbiota is complicated, and little was known about the contribution of different strains to Th17 cell differentiation. In addition, for the known gut microbes and their metabolites involved in the regulation of cell differentiation, the molecular mechanisms of their involvement in regulation also need to be further explored.

### 3.2 Metabolic pathways affect Th17 cell differentiation

Polyamine metabolism, lipid oxidative metabolism, and glycolytic programs are involved in the mediation of naive T cell differentiation (43, 44, 69, 70). For example, the glycolytic pathway regulates Treg differentiation through the transcription factor HIF1α/mTOR signaling pathway, and blocking glycolysis promotes Treg differentiation while inhibiting Th17 development (45, 71). Targeting the glycolytic enzyme glucose phosphate isomerase (Gpi1) to suppress glycolysis is considered to be an effective therapeutic option for Th17-mediated autoimmune diseases with the advantages of minimal toxicity (72). Glutaminase deficiency affects the proliferation and activation of naive T cells and diminishes the differentiation of Th17 cells (73). Lactate has long been considered a waste byproduct of cellular metabolism that accumulates at inflammation sites. Recently, the role of lactate has been redefined as an active metabolite in cell signaling (74, 75). Lactate and the associated hydrion regulate Th17 cell differentiation and are generally considered negative regulators of immunosuppression (75, 76). Under the Warburg effect, tumor cells secrete a large amount of lactate, which enhances TLR ligand-mediated IL-23 expression, resulting in increased IL-17A expression (46). Sodium lactic acid induces the differentiation of CD4^+^ T cells to Th17 cells in rheumatoid arthritis patients; in contrast, lactic acid causes loss of cytolytic function in CD8^+^ T cells (77). These results indicate that the metabolic pathway plays a crucial role in Th17 cell differentiation. However, the possible targets and potential mechanisms of this regulation are not fully understood, and more experimental data are needed to support it.

### 3.3 Role of cytokines in Th17 cell differentiation

IL-6 is an essential molecule that upregulates the expression of the RORγt gene and promotes Th17 cell differentiation *via* activating the JAK2-STAT3 signaling pathway (47). TGF-β is also crucial in Th17 cell differentiation, as it upregulates the expression of the RORγt gene while inhibiting its ability to induce IL-17 expression (78). TGF-β induces Th17 cell differentiation and promotes the emergence of IL-22 production through Smad2 activation, a pathway that can be inhibited by TNF receptor-associated factor 6 (TRAF6) (48). Naive CD4^+^ T cells differentiate into Tregs at high concentration of TGF-β, while, differentiate into Th17 cells at low levels of TGF-β in conjunction with IL-6 (49). Notably, the presence of Th17 cells was not detected in the scarcity of IL-23. IL-23 promotes the stabilization and proliferation of Th17 cells without altering differentiation. In addition, IL-1β not only promotes Th17 polarization *via* the IL1R/PI3K-mTOR signaling pathway but also inhibits TGF-β-induced Foxp3 expression in CD4^+^ T cells (50), thereby inhibiting the differentiation of Tregs (79). Additionally, IL-21 is secreted by Th17 cells, upregulates the expression of IL-17 and RORγt and promotes Th17 cell differentiation through the STAT3 signaling pathway (51).

### 3.4 Regulation of Th17 cell differentiation by transcription factors

Under the induction of cytokines such as IL-6, phosphorylation-activated STAT3 binds to the IL-17 promoter and directly controls the expression of RORγt for Th17 cell differentiation (52). RORγt is a transcription factor that is specific to Th17 cells and directly influences their differentiation (47). Studies have shown that enhanced RORγt promotes Th17 cell differentiation, while RORγt-deficient T cells cannot differentiate into Th17 cells. In addition, IL-17 gene transcription was induced by RORγt (47). B-cell activating transcription factor (ATF-like factor, BATF), JunB, interferon regulatory factor 4 (IRF4), hypoxia inducible factor 1 (HIF1) and basic leucine zipper transcription factor can interfere with Th17 cell differentiation (54–56, 80). IRF4 binds directly to the IL-17 promoter and upregulates the expression of RORγt and IL-17 genes (53). The activator protein 1 (AP-1) transcription factors BATF and JunB are considered essential for Th17 cell differentiation and are considered to be primarily involved in RORγt transcription (54, 55). HIF1a enhances and activates the upregulation of RORγt receptor expression under the glycolytic pathway, thereby increasing the proportion of Th17 cells while inhibiting Foxp3 expression and weakening the differentiation of naive CD4^+^ cells to Tregs (45).

### 3.5 TLR-dependent T-cell differentiation

Activation of the TLR2, TLR3, TLR4, and TLR8 signaling pathways likewise promotes Th17 proliferation, while activation of the TLR7 signaling pathway downregulates STAT3, thereby inhibiting Th17 cell differentiation and IL-17 secretion (57–61). NETs and their histones are mediated downstream of TLR2, resulting in the phosphorylation of STAT3 (57). Activation of TLR2 in DCs mediates IL-23 production. TLR3 and TLR9 respond to pathogen-associated microbial patterns including unmethylated DNA and dsRNA. In the presence of anti-CD3 and anti-CD28, poly (I:C) induces IL-17A and IL-21 synthesis by costimulating naive CD4^+^ T cells through TLR3 (58). Activation of TLR2 in DCs mediates IL-23 production. These results indicated that Th17 cell differentiation and activation are regulated by transcription factor activation and TLR-like receptor signaling.

## 4 The therapeutic potential of IBD by targeting Th17 cells

**Figure 3:**
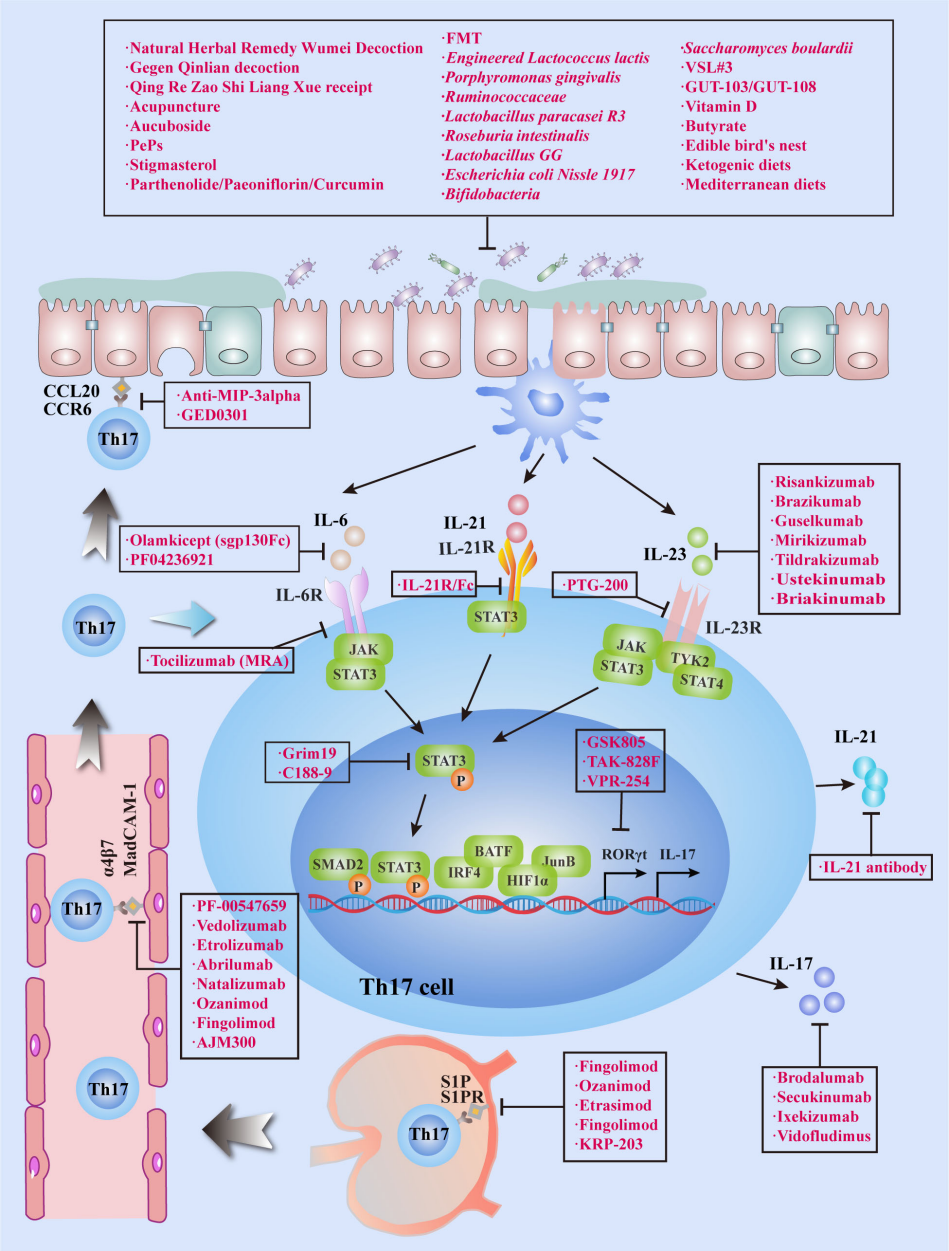
Targeting Th17 cells for treat IBD. IBD can be treated by inhibiting Th17 cell migration, infiltration, differentiation and secretion of cytokines. In addition to conventional treatment methods, medicine or phytochemicals, FMT and probiotics can also inhibit Th17 cell differentiation and IL-17 expression by regulating the bacterial flora, which eventually leads to clinical remission of IBD.

Existing clinical treatments for IBD utilize monoclonal antibodies targeting TNF, and significant progress has been made in this regard, although some patients do not respond to this modality or lose their response over time. Accordingly, IBD requires additional effective and tolerable treatment options. Th17 cells are predominantly located on the surface of the intestinal mucosa and protect the host from the invasion of pathogenic microorganisms.Th17 cells and their secreted cytokines have been shown to play a vital role in IBD pathogenesis, and the imbalance of Treg/Th17 cells can cause IBD. Tregs suppress the excessive immune response of effector T cells and protect intestinal tissue from damage. Some of the existing Th17 cell-related treatment modalities for IBD will be discussed in this section (**Table 2** and **Figure 3**). Above all, the regulation of T-cell differentiation and activation by gut microbiota suggests that a rationally designed bacterial consortium reduces inflammatory cytokines and Th1 and Th17 cells, treats chronic immune-mediated colitis, and restores intestinal homeostasis (100).

**Table 2:** Potential approaches and therapeutic effect of targeting Th17 cells for IBD.

Mechanism	Target	Drug or method	Experimental model or clinical trial status	Effective	Ref
Regulate gut flora	Traditional Chinese medicine therapy	Natural Herbal Remedy Wumei decoction	DSS-induced UC (mice)	YES	(81)
		Gegen Qinlian decoction	DSS-induced UC (mice)	YES	(82)
		Qing Re Zao Shi Liang Xue receipt	TNBS-induced UC (mice)	YES	(83)
		Acupuncture	48-week, randomized, sham controlled, parallel-group clinical trial	YES	(84)
	Plant extract	Aucuboside	TNBS-induced UC (mice)	YES	(85)
		PePs	DSS-induced UC (mice)	YES	(86)
		Stigmasterol	DSS-induced UC (mice)	YES	(87)
		Parthenolide	DSS-induced UC (mice)	YES	(88)
		Paeoniflorin	DSS-induced UC (mice)	YES	(88)
		Curcumin	DSS-induced UC (mice)	YES	(88)
	FMT		Phase II	YES	(89, 90)
	Probiotics	Engineered Lactococcus lactis	DSS-induced UC (mice)	YES	(91)
		Porphyromonas gingivalis	DSS-induced UC (mice)	YES	(92)
		Ruminococcaceae	DSS-induced UC (mice)	YES	(93)
		Lactobacillus paracasei R3	DSS-induced UC (mice)	YES	(94)
		*Roseburia intestinalis*	DSS-induced UC	YES	(95)
		*Lactobacillus GG*	A prospective, randomized, placebo-controlled study	YES	(96)
		*Escherichia coli Nissle 1917*	An open-label pilot study	YES	(97)
		*Bifidobacteria*	A randomized placebo-controlled trial	YES	(98)
		*Saccharomyces boulardii*	Phase II	YES	(99)
		GUT-103	T-cell-transfer IBD (mice)	YES	(100)
		GUT-108	T-cell-transfer IBD (mice)	YES	(100)
		Metabolites of gut microbe	Vitamin D A randomized controlled clinical trial	YES	(101)	
	Butyrate		DSS-induced UC (mice)	YES	(102)
	Diet control	Edible bird’s nest	TNBS-induced UC (rat)	YES	(103)
		Ketogenic diets		YES	(104)
		Mediterranean diets		YES	(105)
Inhibition of Th17 migration	S1P	Fingolimod	DSS-induced UC and TNBS-induced CD (mice)	YES	(106)	
	S1PR	Ozanimod	FDA and EMA approved	YES	(106)
		Etrasimod	Phase II	YES	(107)
		Fingolimod	Phase II	YES	(108)
		KRP-203	A multicenter, double-blind, placebo-controlled, parallel-group study	YES	(109)
	α4β7 integrin	Vedolizumab	FDA approved	YES	(110)
		Etrolizumab	Phase II	YES	(111)
		Abrilumab	Phase II	YES	(112)
		Natalizumab	Phase III	YES	(113)
		Ozanimod	Phase II/III	YES	(106)
		Fingolimod	DSS induced UC (mice)	YES	(111)
		AJM300	A multicentre, randomised, double-blind, placebo-controlled, phase 3 study	YES	(114)
	MAdCAM-1 CCL20	PF-00547659	Phase II	YES	(115)
		Anti-MIP-3alpha	TNBS-induced colitis model (BALB/c mice)	YES	(116)
		Mongersen anti-sense oligonucleotide to Smad7 (GED0301)	TNBS-induced IBD (mice)	YES	(6)
Inhibition of extracellular signal	IL-21R	IL-21R/Fc	DSS-induced UC (mice)	YES	(117)
	IL-6	Olamkicept (sgp130Fc)	Phase II	YES	(118)
		PF04236921	Phase II	YES	(119)
	Il-6R	Tocilizumab (MRA)	Phase II	YES	(120)
	IL-23R	PTG-200	Phase II/I	YES	(121)
	IL-12/23 p40	Ustekinumab	FDA approved	YES	(122)
		Briakinumab	Have stopped production	YES	(123)
	IL-23p19	Risankizumab	Phase III/II	YES	(124)
		Brazikumab	Phase III/II	YES	(125)
		Guselkumab	Phase III/II	YES	(126)
		Mirikizumab	Phase III/II	YES	(127)
		Tildrakizumab	Phase III/II	YES	(126)
Inhibition of nuclear transcription	RORγt	GSK805	Citrobacter rodentium (mice)	YES	(128)
		TAK-828F	T-cell-transfer IBD (mice)	YES	(129)
		VPR-254	DSS, TNBS and anti-CD40 antibody-induced murine models of colitis (mice)	YES	(130)
	STAT3	Grim19	DSS-induced colitis mouse model	YES	(131)
		C188-9	DSS-induced UC and TNBS-induced CD (mice)	YES	(132)
Inhibition of effector cytokine	IL-17R	Brodalumab	Phase II	NO	(133)
	IL-17A	Secukinumab	A randomized, double blind, placebo-controlled trial	NO	(134)
		Ixekizumab	Phase III	NO	(135)
	IL-17A/IL-17F	Vidofludimus	Phase I	YES	(136)

### 4.1 Regulating gut microbiota inhibits Th17 cell differentiation and activation

*In vivo*, a complex microenvironment of commensal flora drives IL-23 production by DCs in the ileum propria and CCL20 expression by IECs. *SFB, Escherichia coli *O157, and *Citrobacter rodentium *are examples of bacteria that attach to IECs and selectively induce Th17 responses in an IL-23-dependent manner. (64, 65). The treatment of IBD by regulating gut microbes is effective and has become a research hotspot in recent years; its main mode of action is to regulate the recruitment, differentiation and activation of Th17 cells in the intestinal mucosal microenvironment.

Traditional Chinese medicine therapy is usually characterized by safe and remarkable therapeutic effects. Chinese medicinal herbal decoctions, such as Natural Herbal Remedy Wumei decoction, Gegen Qinlian decoction and Qing Re Zao Shi Liang Xue receipt, ameliorate intestinal mucosal inflammation by maintaining microbial homeostasis and restoring Th17/Tregs balance. These decoctions change the diversity and community landscape of the gut microbiota and metabolic profiles and in particular significantly increased the abundance of SCFAs produced by the gut microbiota (81–83). Similarly, the relative abundance of *Roseburia faecis* and *Faecalibacterium prausnitzii* in gut microbiota was increased, and the expression of Th17 cell-related cytokines was decreased in CD patients treated with acupuncture (84).

Plant extract is an effective preparation for the treatment of IBD. Aucuboside is a compound extracted from traditional Chinese medicine that significantly inhibits the production of Th17 cells in colitis, accompanied by the inhibition of IL-17 expression (85). Periploca sepium periplosides (PePs) are cardiosinole-free progestane glycosides derived from the rhizocarp of rhizocarp, changes the gut microbes and plays a vital role in regulating intestinal Th17 cell immunity (86). Stigmasterol regulates the gut microbes of mice with enteritis and increases butyric acid-producing bacteria. Butyrate can promote Treg differentiation, inhibit Th17 differentiation, and reduce colonic inflammation in experimental mice (87). Parthenolide, paeoniflorin, curcumin, 6-gingerol, tripterygium wilfords polyglycoside, baicalein, baicalin and rhubarb peony decoction improve colonic inflammation by regulating the Treg/Th17 balance via the gut microbes (88).

In recent years, some researchers have also proposed that FMT can be used to reconstruct gut microbes and have clinically proven its effectiveness in the treatment of IBD (89, 90, 137, 138). The high cost of FMT led researchers to propose the concept of "precise capsules". In our previous study, *Roseburia intestinalis* was shown to reduce inflammation in animal models (95). After treating IBD mice with *Porphyromonas gingivalis*, *Lactobacillus rhamnosus* GG, *Ruminococcaceae* or *Lactobacillus paracasei* R3, intestinal inflammation was relieved, where Tregs increased significantly in the intestinal tissue, while Th17 cells decreased, and intestinal inflammation was relieved (92–94). Engineered *Lactococcus lactis*, *Escherichia coli Nissle 1917*, *Bifidobacteria* and *Saccharomyces boulardii* strains have achieved good effects in clinical research of IBD by regulating intestinal microecology (91, 97–99). GUT-103 and GUT-108, which are composed of 8, 17 and 11 intestinal bacteria, respectively, are used to treat IBD and mediate the reduction of inflammatory factors and Th17 cells (100). Unfortunately, the molecular mechanism of other functional strains in the treatment of IBD has not been further investigated. In recent years, although the interaction between the gut microbiota and the host has been gradually revealed in the progression of IBD, the mechanism needs to be further elucidated.

Vitamin D, SCFAs, and metabolites of gut microbes have anti-inflammatory properties that protect the intestinal epithelium, including promoting Treg cell differentiation and upregulating the expression of anti-inflammatory factors (101, 102). Nutrient intake is one of the most important factors affecting gut microbes and intestinal mucosal immunity. For example, in a pilot study in humans subjects, a moderate high-salt challenge increased Th17 cells by reducing intestinal survival of *Lactobacillus* spp. Excessive sugar intake mediates the increase in the cytokines IL-1β and Th17 cells, which induce inflammation (139). Edible bird's nest can recuperate dextran sulphate sodium (DSS)-induced UC in C57BL/6J mice by recovering the Th17/Treg cell balance (103). Ketogenic and Mediterranean diets decrease gut Th17 cells by altering the gut microbiome (104, 105). Therefore, diet control is of great significance in the adjuvant treatment of IBD.

In addition to the abovementioned treatments, OPS-2071 is a quinolone antibiotic used to treat intestinal infections with low-absorption properties, dose-dependent reduction of colitis histological score and colonic weight to length ratio in a mouse model (140). Mesenchymal stem cells may alleviate IBD by regulating Treg/Th17 cell differentiation (141), thus providing a new option for IBD treatment. Due to the unknown molecular mechanism of their regulation of Treg/Th17 cells, we will not go into detail here.

### 4.2 Inhibition of Th17 cell migration

In lymphatic endothelial cells, the sphingosine 1-phosphate receptor (S1PR) is highly expressed. Th17 cells migrate through the sphingosine 1-phosphate (S1P) -S1PR axis in lymphatic vessels. Therefore, targeting regulation of the S1P-S1PR axis to inhibit Th17 cell migration is a potential therapeutic approach for IBD. The S1P modulator fingolimod has been shown to be effective in animal models of enteritis (106). Modulators of S1PR, including ozanimod, etrasimod, ponvory (ponesimod), and KRP-203, have achieved good clinical efficacy (106–109).

Adhesion and infiltration of Th17 cells depend on the binding of α4β7 integrin to the adhesion molecule MAdCAM-1. Anti-α4β7 integrin antibodies relieve local inflammatory responses by blocking Th17 cell migration to inflammation site. Vedolizumab, etrolizumab, abrilumab, and natalizumab are some of the reported anti-α4β7 integrin antibodies that have shown good safety and efficacy in the treatment of IBD (110–113). Natalizumab and vedolizumab are FDA-approved for the therapy of moderate-to-severe IBD. Etrolizumab was shown to alleviate UC inflammation although the effectiveness is but was not better than adalimumab (111). Abrilumab is associated with extremely high remission, clinical response, and mucosal healing rates in patients with moderate-to-severe UC. In addition, other molecules with therapeutic potential are α4β7-specific ozanimod, fingolimod, and AJM300 (106, 111, 114). PF-00547659, an antibody target endothelial MAdCAM-1, has also shown therapeutic potential for UC but not for CD (115).

The homing and migration of immune cells depend on the expression of chemokine receptors. Macrophage inflammatory protein (MIP)-3α is elevated in IBD is considered to be a CD4 T-cell directed chemokine. Anti-MIP-3alpha significantly reduced T-cell recruitment and TNBS-mediated colonic injury (116). CCL20 is a CCR6 homologue ligand with strong affinity. Th17 cells have strong CCR6 expression, which promotes their migration to inflammatory tissues with high levels of CCL20. Mongersen-anti-sense oligonucleotide to Smad7 (GED0301) is a short oligonucleotide that indirectly targets CCL20 and alleviates inflammation in a TNBS-induced mouse model of IBD (6).

### 4.3 Inhibiting Th17 cell proliferation and differentiation

Th17 cell differentiation, proliferation, and activation are induced by a variety of cytokines, such as IL-6, IL-21, and IL-23. Neutralization of these cytokines or inhibition of their receptors by monoclonal antibodies or small molecule inhibitors can inhibit Th17 cell production and achieve IBD therapy. In animal models, recombinant anti-IL-6 antibodies can alleviate symptoms of colonic inflammation by hindering the secretion of inflammatory factors, reducing inflammatory cell aggregation, and promoting T-cell apoptosis (118, 119). PF04236921 is a human IL-6 monoclonal antibody. A clinical study involving 247 patients with IBD showed that the clinical response rate and remission rate of the treatment group taking the drug were better than those of the placebo group, and there was a certain dose-effect relationship (119). Tocilizumab (MRA), a humanized monoclonal antibody directed against the IL-6 receptor, showed promising efficacy in patients with active CD (120).

Studies found that after the administration of the recombinant protein IL-22 to mice with colitis, symptoms and intestinal inflammation were relieved, but there are no reports on the clinical application of IL-22 (28). Anti-TNFα preparations upregulate the ratio of Tregs versus effector T cells, mainly by reducing the expression of inflammatory factors (including Th17-cell-related IL-17, IL-23 and IL-6), increasing the number of Tregs, and promoting effector T-cell apoptosis. The specific binding of infliximab to TNFα can block the binding of the latter to cell surface receptors, thereby inhibiting the inflammatory response and rapidly inducing apoptosis of intestinal mucosa T cells. However, the drug is only effective for some patients; its efficacy may be related to the type of IBD and various inflammatory cytokines.

Ustekinumab, an antibody that blocks IL-12 and IL-23, has been greenlighted for the treatment of CD and UC (122). Briakinumab, another antibody that inhibits IL-23 and IL-12 in combination, is effective against IBD although it has been discontinued (123). Several anti-IL-23p19-specific antibodies have undergone or are presently undertaking clinical studies for IBD, such as brazikumab, risankizumab, mirikizumab, and guselkumab (124–127). PTG-200 is an IL-23R antagonists therefore also specifically interfere with IL-23 signaling (121).

In addition to targeting cytokines associated with mediating Th17 cell differentiation, the master transcriptional regulator RORγt itself is an intriguing therapeutic target that may have impacts not only on Th17 cells but also on type 17 cells generally. It targets cytokines linked to facilitating Th17 cell development. The RORγt antagonists VPR-254, GSK805, and TAK-828F, which have shown promise efficacy in preclinical models of IBD, are among those now being developed (128–130). The underlying mechanisms of action, important target cells (such as Th17 cells *versus* ILC3s), and impacts on the homeostatic balance between Th17 cells and Tregs are all unanswered problems involving RORγt antagonism. In addition, the transcription factor STAT3 can also be used as a target to inhibit Th17. STAT3-related inhibitors, such as gene associated with retinoid interferon-induced mortality (Grim) 19, metformin and C188-9, have shown good therapeutic effects in murine models of CD and UC (131, 132).

### 4.4 Neutralization or inhibition of cytokines produced by Th17 cells

Neutralizing antibodies and small molecule inhibitors targeting Th17 effector molecules can exhibit different therapeutic effects in different enteritis states. Although IL-17 is overexpressed in IBD patients, the IL-17A neutralizing antibody (secukinumab and ixekizumab) and the IL-17A receptor neutralizing antibody (brodalumab) are not effective in CD patients, and some patients have adverse reactions (133–135). Wedebye Schmidt et al. discovered that simultaneous inhibition of IL-17A and IL-17F expression can effectively alleviate T-cell transplantation-induced intestinal inflammation in mice (142). Vidofludimus is an oral immunosuppressant that can downregulate IL-17A and IL-17F levels by inhibiting STAT3 and NF-κB, demonstrating safety, efficacy and tolerability in hormone-dependent IBD patients (136). These results suggested that combined therapy and targets multiple cytokines is expected to become a new option for IBD treatment. Clinical trials have confirmed that secukinumab, ixekizumab, and brodalumab can effectively alleviate various diseases, such as multiple sclerosis (MS), active ankylosing spondylitis (AS), rheumatoid arthritis (RA) or psoriasis, and are ineffective in the treatment of CD and UC (133–135). IL-21 receptor blockers alleviate DSS-induced colitis in mice (117), indicating that IL-21 may also be a potential target for the treatment of IBD in the future.

## 5 Conclusion

Unlike other autoimmune diseases, the pathogenesis of IBD occurs in the gut, where the largest proportion of intestinal symbiotes and intestinal microbes are colonized. Through the review of Th17 cell immunity, we have gained more understanding of the pathogenesis and treatment of IBD. Th17 cells are potently affected by the gut microecosystem and are fully involved in the disease progression of IBD from intestinal homeostasis to inflammation to fibrosis. In recent years, a growing number of studies have shown that the gut microbes were involved in intestinal homeostasis and IBD by regulating Th17 cell differentiation and activation. Traditional Chinese medicine decoction, plant extract and gut microbes have attracted tremendous interest in the treatment of IBD through the gut microbes-Th17 cell axis, which is safe, effective and has few side effects, providing more possibilities for a better prognosis of IBD. These therapies complement the traditional Th17 cell-targeted immunobiologics for IBD. Unfortunately, the existing findings do not fully explain the immunomodulatory mechanisms of the gut microbiome. More evidence was needed to evaluate the contribution of gut microbes to mucosal immunity.

## 6 Perspective

Previous studies have shown that Th17 cells and their secreted cytokines play a crucial role in the occurrence and development of IBD and serve as a bridge between gut microbes and the gut immune microenvironment information. Theoretically, the superiority of Th17-targeted immunotherapy lies in alleviating IBD inflammation while improving intestinal fibrosis. The ineffectiveness of secukinumab, ixekizumab and brodalumab in the treatment of CD patients and the development of resistance after long-term biologic treatment suggest that the molecular mechanism of Th17 cells in IBD needs to be further investigated. We speculate that the diversity of intestinal microbes increases the complexity of the mucosal microenvironment and provides more possibilities for interaction between Th17 cells and other immune cells, making the situation in other tissues and organs not applicable to the intestinal microenvironment.

In the future, the in-depth study of gut microbes and Th17 cells in IBD, it will be of great help to the diagnosis, disease status assessment, drug efficacy and prognosis evaluation of IBD. Adjustment of intestinal microecology, selection of specific microorganisms and FMT combined with biological therapies may revolutionize the treatment of IBD and ultimately improve patient prognosis.

## Author contributions

LC, DC, and YW wrote the manuscript. GR, YC, and AY conducted the literature survey. LC generated themes and ideas and edited the manuscript. This work was supervised by DC and YW. All authors contributed to the article and approved the submitted version.
